# Older adult patients with out-of-hospital pressure injuries: a single-center retrospective study in a tertiary hospital in China

**DOI:** 10.3389/fmed.2026.1859691

**Published:** 2026-08-25

**Authors:** Hongling Sun, Caiyun Xu, Yan Sun, Hui Wang, Shuyan Li, Jinping Xia

**Affiliations:** Department of Nursing, The Second Affiliated Hospital, Zhejiang University School of Medicine, Hangzhou, China

**Keywords:** community-acquired pressure injury, older adults, influencing factors, out-of-hospital, pressure injury, treatment outcomes

## Abstract

**Background:**

To investigate the prevalence and clinical characteristics of pressure injuries (PIs) present at hospital admission among older adult patients, and to identify factors influencing healing outcomes through a single-center retrospective analysis. The goal is to inform nursing strategies for PI management in older adults.

**Methods:**

Data were retrieved from a self-developed pressure injury prevention and management platform at a tertiary hospital in Zhejiang Province. Patients aged ≥60 years admitted between January 2022 and December 2023 with preexisting PIs were included. Data on demographics, medical conditions, PI onset timing, staging, and outcomes were collected. Univariate analysis was performed using healing status as the outcome variable, followed by logistic regression to identify associated factors.

**Results:**

In total, 30,811 older adult patients with PIs were included, representing 10.89% of all admissions. Males accounted for 63% and females 37%. In 85% of cases, PIs originated in the home, and 71.80% involved multiple sites. Only 44 patients (0.14%) were admitted specifically for PI treatment. The sacrococcygeal region was the most common site (22.67%). Overall, 49.08% of cases were Stage 1 PIs, while 41.70% were Stage 3 or higher. The overall healing rate was 21.90%. Multivariate regression analysis identified sex, disease type, PI onset timing, Braden score, and hospital length of stay as significant predictors of healing outcomes.

**Conclusion:**

The prevalence of preexisting PIs among older adult patients admitted to tertiary hospitals is high, with delayed treatment and limited awareness among home caregivers. Strengthening wound care and preventive quality management through hospital-based platforms may be associated with improve healing outcomes. There is an urgent need for post-discharge care and education. Leveraging tertiary hospital expertise to deliver internet-based nursing services or empower primary care providers in PI prevention and wound care is suggested as a potential strategy to enhance quality of life for older adult patients in home and rehabilitation settings. However, these hypotheses require validation through prospective interventional studies.

## Background

Pressure injury (PI) refers to localised damage to the skin and/or underlying soft tissue caused by sustained pressure, or a combination of pressure and shear force. Out-of-hospital PI, also known as community-acquired pressure injury (CAPI), is defined as an injury present prior to a patient’s admission to a healthcare facility (1). Globally, the incidence of PI among hospitalised patients is as high as 12.8% (2). In out-of-hospital settings, this rate can reach up to 48.0% (3), and in home care environments, it remains substantial at 29.0% (4), underscoring the severity of the issue.

PI has a profound impact on quality of life. It prolongs hospital stays, increases healthcare costs (5), and contributes to psychological distress. Moreover, PIs are prone to secondary infections that may lead to severe complications such as sepsis, and in some cases can be life-threatening (6). With the aging population steadily increasing, the proportion of older adult inpatients at our hospital increased from 43.8% in 2022 to 47.2% in 2023. Among these patients, 10.89% were admitted with pre-existing PI.

Understanding the clinical characteristics and outcome-related factors of out-of-hospital PI in older adult patients is essential for improving care strategies. This study retrospectively analysed data from 30,811 older adult patients with out-of-hospital PI to identify key factors influencing outcomes.

## Materials and methods

### General data

A retrospective investigation was conducted on the clinical data of older adult patients with out-of-hospital PI reported by various departments in our hospital’s information system from January 2022 to December 2023, with a total of 30,811 cases included (total *n* = 1,413,570). Inclusion criteria were as follows: age ≥60 years; PI diagnosis consistent with the diagnostic criteria outlined in the International Pressure Ulcer Prevention and Treatment Guidelines (2019 Edition); confirmed characteristics of out-of-hospital PI, and informed consent obtained from the patient or family. Exclusion criteria included incomplete clinical data and a hospital stay of 48 h or less. Data were retrieved from a self-developed pressure injury prevention and management platform at a tertiary hospital in Zhejiang Province, China. This hospital has approximately 3,44,000 annual inpatient admissions and discharges, of which over 60% are older adult patients, and its intensive care units have more than 200 beds. To avoid duplicate counting, we performed strict deduplication based on patient identification information through the hospital’s Medical Records Department system, excluding repeated admissions of the same patient. All cases were verified by specialized staff from the Medical Records Department based on inpatient medical records and admission skin assessment documentation. A total of 30,811 eligible older adult patients were identified and included in this study.

Out-of-hospital PI was defined as a pressure injury present on admission (POA) that occurred prior to the current hospitalization in out-of-hospital settings (e.g., at home, other hospitals, or nursing homes). Following admission, the responsible nurse completed a Braden Scale risk assessment and a full-body skin examination within 24 h. If an out-of-hospital PI was identified or the Braden score was ≤12, the ward PI specialist nurse completed an online “Pressure injury/High-risk patient notification form” with mandatory classification of the case as “out-of-hospital PI” or “high-risk”. The system then automatically reported and categorized the case. A core team member (head nurse from high-risk units) conducted an on-site primary verification within 48 h to confirm the PI stage and assessment accuracy. Subsequently, a full-time specialist nurse (international enterostomal therapist) performed a secondary verification within 24 h (holidays excluded). The verification results and recommendations were recorded in the electronic review system, and the ward implemented or adjusted nursing measures accordingly. The full-time specialist nurse and team leader conducted weekly on-site inspections of all patients with out-of-hospital PIs, with dynamic documentation of progression until discharge or outcome closure. All out-of-hospital PI cases were archived with blue classification (distinguished from red for hospital-acquired PIs), and monthly statistics were reported to the Nursing Department. All data were cross-validated by specialized staff from the Medical Records Department based on inpatient medical records and admission skin assessment documentation, ensuring the authenticity, accuracy, and reliability of the data.

### Methods

The wound and stoma care team collected relevant data on older adult patients with out-of-hospital PI, including sex, age, department, diagnosis, time and place of occurrence, Braden score, outcome, length of hospital stay, PI location, stage, and size. PI staging was determined according to the NPUAP/EPUAP 2019 Pressure Injury Staging System. Deep tissue injury (DTI) was defined as a persistent non-blanchable deep red, maroon, or purple discoloration of intact or non-intact skin, or epidermal separation revealing a dark wound bed or congested blister. Unstageable PI was defined as full-thickness skin or tissue loss in which the actual depth of the ulcer is obscured by slough or eschar in the wound bed. All staging assessments were initially performed by the responsible ward nurse upon admission and subsequently verified by core team members (head nurses from high-risk units) within 48 h and by full-time specialist nurses (international enterostomal therapists) within 24 h to ensure accuracy. The Braden scale consists of six factors: friction and shear, sensation, moisture, mobility, activity, and nutrition (7). The total score ranges from 6 to 23, with lower scores indicating a higher risk of PI. Scores of 15–18 indicate mild risk, 13–14 indicate moderate risk, 10–12 indicate high risk, and 6–9 indicate very high risk. At discharge, the PI condition was assessed and compared to the stage at admission to determine the outcome, which was categorised as healed, improved, no progress, or worsened. Improved, no progress, and worsened were collectively defined as “non-healed”. We analysed the differences between healed and non-healed cases. Length of hospital stay was categorized based on clinically meaningful thresholds commonly used in practice: ≤7 days (short-stay), 8–14 days (medium-stay), 15–30 days (prolonged-stay), 31–60 days, 61–90 days, and >90 days (extended-stay). This categorization facilitates clinical interpretation and practical application of the findings. This retrospective study was approved by the Ethics Committee of the Second Affiliated Hospital, School of Medicine, Zhejiang University (Hangzhou, Zhejiang Province, China) (approval No. 2025-1544). The clinical data included in this study originated from routine medical records generated during the period of January 2022 to December 2023 as part of standard clinical practice, not as a prospective research activity. Formal ethical approval was obtained in early 2025, and all research activities involving human data-including data extraction, anonymization, statistical analysis, and manuscript preparation-were conducted strictly after the approval was granted. The Ethics Committee also granted a waiver of informed consent, as this study involved only anonymized retrospective analysis of existing medical records without any additional patient contact or intervention, and posed no more than minimal risk to patients.

### Statistical methods

Data were entered into Excel software and analysed using SPSS 25.0 statistical software. Descriptive statistics are presented as counts and percentages (%). Normally distributed continuous data are expressed as mean ± standard deviation (*x* ± *s*), and categorical data are presented as percentages (%). Chi-square (χ^2^) tests were used for comparisons between two independent samples, and chi-square tests for R × C tables were used for multiple samples. For variable selection, a two-step approach was employed: univariate analysis was first performed, and variables with *p* < 0.1 were entered into the multivariable model; forward stepwise selection was then applied for multivariable logistic regression, with entry criterion set at *p* < 0.05 and removal criterion at *p* > 0.10, to identify independent influencing factors. The reference categories for categorical variables were set as follows: male (for sex), cancer (for type of disease), ≤1 week (for time of PI occurrence), 6–9 (for Braden score), ≤7 days (for length of hospital stay), and single PI (for occurrence of PIs). Missing data were handled by listwise deletion, with a missing proportion of less than 1% for core variables. Multivariate logistic regression analysis was employed to identify factors influencing the outcomes of out-of-hospital PI. A *p*-value <0.05 was considered statistically significant.

## Results

### General information of older adult patients with out-of-hospital PI

Among the 30,811 older adult patients with out-of-hospital PI, the age ranged from 60 to 104 years, with an average age of 74.79 ± 8.65 years. The average Braden score was 15.89 ± 2.73, and the average length of hospital stay was 10.40 ± 12.75 days. The details are shown in Table 1.

**Table 1:** Basic information of older adults patients with out-of-hospital PI.

Characteristics		Number	Proportion
Sex	Male	19,420	63.00%
	Female	11,391	37.00%
Age (y)	60–74	16,038	52.10%
	75–89	13,113	42.60%
	≥90	1,660	5.40%
Type of disease	Cancer	5,956	19.30%
	Trauma/Orthopaedic	4,924	16.00%
	Cardiovascular	3,575	11.60%
	Cerebrovascular	3,093	10.00%
	Respiratory	2,735	8.90%
	Diabetes	385	1.20%
	Other	10,143	32.90%
Time of occurrence	≤1 week	13,130	42.61%
	1 week–1 month	11,820	38.36%
	1–3 months	2,685	8.70%
	3–6 months	1,796	5.80%
	6 months–1 year	537	1.70%
	>1 year	843	2.70%
Braden score	6–9	191	0.60%
	10–12	5,340	17.30%
	13–14	3,408	11.10%
	15–18	19,187	62.30%
	19–23	2,685	8.70%
Place of occurrence	Home	26,019	84.40%
	Other hospitals	4,427	14.40%
	Nursing home	365	1.20%
Length of stay (days)	≤7	15,739	51.10%
	8–14	9,029	29.30%
	15–30	4,804	15.60%
	31–60	1,037	3.40%
	61–90	131	0.40%
	>90	71	0.20%
Single or multiple	Single	8,676	28.20%
	Multiple	22,135	71.80%
Clinical outcome	Healed	6,743	21.90%
	Improved	14,772	47.90%
	No Progress	9,295	30.20%
	Worsened	1	0.00%

### Basic information of PI locations in older adult patients

**Figure 1:**
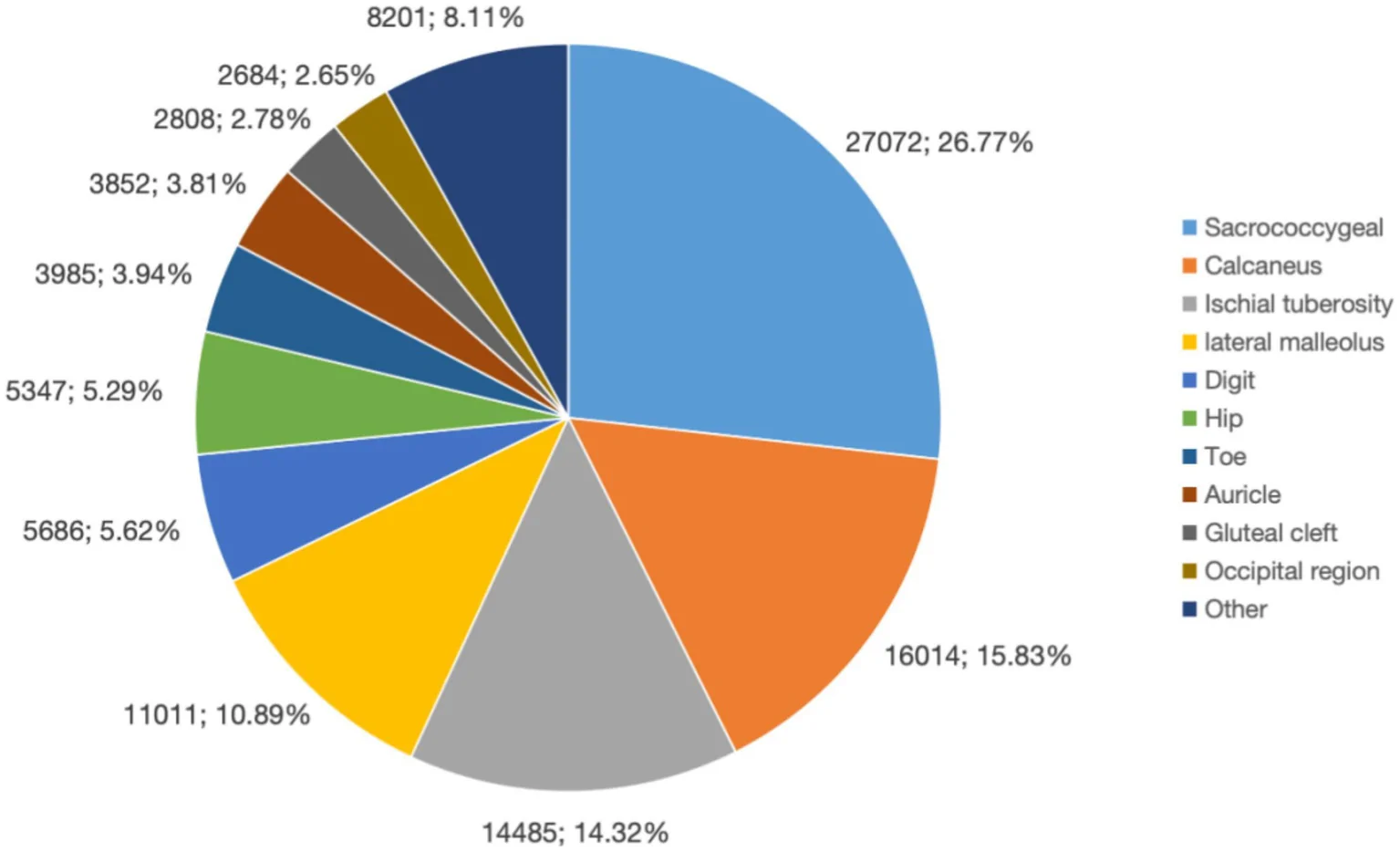
PI locations in older adults patients.

The most common locations for out-of-hospital PI were the sacrococcygeal area, heel, and ischial tuberosity, accounting for 26.77, 15.83, and 14.32% of cases, respectively. Details are given in Figure 1.

### Staging of PI in older adult patients

The majority of out-of-hospital PIs in older adult patients were Stage 1, accounting for 49.08% of the total. Suspected deep tissue injury and Stage 2 PIs accounted for 35.88 and 9.22%, respectively. The details are shown in Table 2.

**Table 2:** Staging distribution of out-of-hospital PI.

Stage	No. sites	Proportion (%)
Deep tissue injury	36,294	35.88%
Unstageable	5,376	5.32%
I	49,642	49.08%
II	9,330	9.22%
III	322	0.32%
IV	183	0.18%

### Univariate analysis of outcomes for older adult patients with out-of-hospital PIs

Univariate analyses indicated that sex, disease diagnosis, time of occurrence, Braden score, length of hospital stay, and the specifics of PI occurrence influenced the outcomes of out-of-hospital PIs in older adult patients, with statistically significant differences (*p* < 0.05). The detailed results are shown in Table 3.

**Table 3:** Univariate analysis of outcomes for out-of-hospital PI.

Items	Healed (n=6743)	Unhealed (*n* = 24,068)	*x* ^2^	*p* value
Sex			89.843	<0.001
Male	3,918 (58.10%)	15,502 (64.41%)		
Female	2,825 (41.90%)	8,566 (35.59%)		
Age (y)			5.185	0.075
60–74	3,591 (53.26%)	12,447 (51.72%)		
75–89	2,791 (41.39%)	10,322 (42.89%)		
≥90	361 (5.35%)	1,299 (5.40%)		
Diagnosis			1714.626	<0.001
Cancer	649 (9.62%)	5,307 (22.05%)		
Diabetes	148 (2.19%)	237 (0.98%)		
Cerebrovascular	688 (10.20%)	2,405 (9.99%)		
Cardiovascular	839 (12.44%)	2,736 (11.37%)		
Trauma/Orthopaedic	2021 (29.97%)	2,903 (12.06%)		
Respiratory	328 (4.86%)	2,407 (10.00%)		
Others	2070 (30.70%)	8,073 (33.54%)		
Time of occurrence			692.177	<0.001
≤1 week	3,807 (56.46%)	9,323 (38.74%)		
1 week–1 month	2,042 (30.28%)	9,778 (40.63%)		
1–3 months	418 (6.20%)	2,267 (9.42%)		
3–6 months	297 (4.40%)	1,499 (6.23%)		
6 months–1 year	73 (1.08%)	464 (1.93%)		
>1 year	106 (1.57%)	737 (3.06%)		
Braden score			80.162	<0.001
6–9	22 (%)	169 (%)		
10–12	965 (%)	4,375 (%)		
13–14	748 (%)	2,660 (%)		
15–18	4,331 (%)	14,856 (%)		
19–23	677 (%)	2,008 (%)		
Source			2.678	0.262
Home	5,665 (%)	20,354 (%)		
Other hospitals	1,005 (%)	3,422 (%)		
Nursing home	73 (%)	292 (%)		
Length of stay (days)			54.502	<0.001
≤7	3,266 (48.44%)	12,473 (51.82%)		
8–14	2,218 (32.89%)	6,811 (28.30%)		
15–30	1,007 (14.93%)	3,797 (15.78%)		
31–60	208 (3.08%)	829 (3.44%)		
61–90	27 (0.40%)	104 (0.43%)		
>90	17 (0.25%)	54 (0.22%)		
Single or multiple			146.615	<0.001
Single PI	2,294 (34.02%)	6,382 (26.52%)		
Multiple PI	4,449 (65.98%)	17,686 (73.48%)		

### Multivariate analysis of outcomes for older adult patients with out-of-hospital PI

Taking the outcome of PI at discharge (non-healed = 0, healed = 1) as the dependent variable, multivariate logistic regression analysis was conducted using the independent variables that were statistically significant in univariate analyses. The results indicated that sex, disease diagnosis, time of occurrence, Braden score, length of hospital stay, and the specifics of PI occurrence were the main factors influencing the outcomes of out-of-hospital PI in older adult patients. The results for each independent variable are detailed in Table 4 and described below.

**Table 4:** Multivariate analysis of outcomes for out-of-hospital PIs.

Items	*β*	Std	Wald χ^2^	*p* value	OR (95% CI)
	−2.643	0.238	123.821	<0.001	
Sex	0.066	0.030	4.945	0.026	1.068 (1.008–1.132)
Type of disease					
Diabetes	1.630	0.115	201.491	<0.001	5.102 (4.074–6.390)
Cerebrovascular	1.006	0.065	239.064	<0.001	2.734 (2.407–3.106)
Cardiovascular	1.011	0.059	297.926	<0.001	2.749 (2.450–3.083)
Trauma/orthopaedic	1.687	0.054	981.129	<0.001	5.402 (4.861–6.004)
Respiratory	0.282	0.074	14.640	<0.001	1.326 (1.147–1.531)
Others	0.806	0.049	266.760	<0.001	2.240 (2.033–2.468)
Time of occurrence					
1 week–1 month	−0.505	0.034	226.587	<0.001	0.604 (0.565–0.645)
1–3 months	−0.606	0.059	103.874	<0.001	0.546 (0.486–0.613)
3–6 months	−0.549	0.069	62.476	<0.001	0.577 (0.504–0.662)
6 months–1 year	−0.788	0.130	36.544	<0.001	0.455 (0.352–0.587)
≥1 year	−0.878	0.109	65.395	<0.001	0.415 (0.336–0.514)
Braden score					
13–14	0.753	0.234	10.350	0.001	2.124 (1.342–3.360)
15–18	1.035	0.232	19.958	<0.001	2.815 (1.788–4.432)
19–23	1.327	0.236	31.515	<0.001	3.769 (2.372–5.991)
Length of stay (days)					
8–14	0.162	0.033	23.652	<0.001	1.175 (1.101–1.255)
15–30	0.122	0.043	7.894	0.005	1.129 (1.037–1.229)
>90	0.583	0.292	3.990	0.046	1.792 (1.011–3.176)
Occurrence of PI					
Multiple PI	−0.355	0.032	126.08	<0.001	0.701 (0.659–0.746)

Reference categories for categorical variables were set as follows: male (for sex); cancer (for type of disease); ≤1 week (for time of PI occurrence); Braden score 6–9 (for Braden score); ≤7 days (for length of hospital stay); and single PI (for occurrence of PIs). These reference categories were selected based on clinical practice standards, representing either the highest-risk group (e.g., cancer, Braden 6–9) or the most clinically common category (e.g., ≤1 week, ≤7 days) to facilitate clinical interpretation.

Among disease categories, patients with diabetes (OR = 5.102, 95% CI: 4.074–6.390) and trauma/orthopedic conditions (OR = 5.402, 95% CI: 4.861–6.004) exhibited the highest odds of healing, while patients with cancer (reference category) showed the lowest healing rates. For time of occurrence, PIs occurring within 1 week prior to admission were associated with the highest healing rates (reference), with progressively lower odds observed for longer durations. Higher Braden scores were consistently associated with better healing outcomes. Extended hospital stays (8–14 days, 15–30 days, and >90 days) showed modestly increased odds of healing compared to stays ≤7 days. Multiple PIs were associated with significantly lower odds of healing (OR = 0.701, 95% CI: 0.659–0.746) compared to single PIs. Female patients exhibited slightly higher odds of healing (OR = 1.068, 95% CI: 1.008–1.132) compared to males.

## Discussion

### Prevention of out-of-hospital PIs in older adult patients is challenging and requires attention

In all, 30,811 older adult patients with out-of-hospital PIs were included in this study, with 1,01,145 PIs recorded, averaging 3.28 per person. This incidence rate is significantly higher than that reported in previous studies (8, 9). More than 70% of the older adult patients had multiple injuries, which may be attributable to the fact that most of the included patients were from high-risk groups, such as those with tumours, cardiovascular and cerebrovascular diseases, respiratory system diseases, and orthopaedic trauma. These patients often present with risk factors including prolonged bed rest, limited mobility, sensory impairment, inadequate tissue perfusion, malnutrition, and the use of orthopaedic devices, all of which may contribute to the increased incidence of PIs.

Among the included patients, the number of males was approximately 1.70 times that of females, consistent with previous findings (6). This may be due to the generally lower percentage of body fat in males, which reduces the natural cushioning at pressure points and increases the risk of skin and subcutaneous tissue damage under sustained pressure or shear force.

The locations of PI occurrence included the home, other hospitals, and nursing homes. Overall, 84.40% of the patients had been injured at home, consistent with previous studies (9, 10). For such patients, rehabilitation is primarily managed by family members and caregivers, who often lack professional knowledge and skills in PI prevention and treatment. In addition, the absence of necessary pressure-relief devices may further increase the risk of PIs.

In terms of staging, Stage 1 accounted for the majority (49.08%) of PIs. However, a substantial proportion of patients presented with severe PIs upon admission, accounting for 41.7% (deep tissue injury 35.88%, unstageable 5.32%, Stage III 0.32%, Stage IV 0.18%). It should be noted that deep tissue injury (DTI) does not always equate to irreversible tissue damage; with timely and effective offloading and supportive interventions, some DTI cases may be reversible. Nevertheless, in this retrospective cohort, all DTI cases were already present on admission, and their potential for reversal could not be determined from available data. The average length of hospital stay in 2023 was 5.7 days, while the average stay for older adult patients with out-of-hospital PIs was 10.40 ± 12.75 days. PI severity may contribute factor to delayed recovery and prolonged hospitalisation.

The most common sites of out-of-hospital PIs were the sacrococcygeal area, heel, and ischial tuberosity, consistent with findings from previous studies (9, 11, 12). These areas are frequent pressure points during body position changes, and the skin tissue in these regions is relatively thin with reduced blood circulation, making them critical targets for pressure relief and prevention.

PI poses a significant threat to quality of life. It not only prolongs hospital stays but also increases medical resource consumption and the economic burden on patients. Furthermore, PIs exacerbate physical and psychological stress, increase the risk of secondary infections, and can directly endanger patient safety. Therefore, prevention, early identification, and timely treatment are essential to mitigate their adverse effects.

In summary, the incidence of PIs in older adult patients with out-of-hospital PIs is high, and the issues of neglect and delayed treatment remain serious. These challenges require urgent attention, and treatment should be actively tailored to individual patient conditions. Simultaneously, health education for patients and their families should be strengthened.

### Factors influencing the treatment outcomes of out-of-hospital PIs

Sex, type of disease, time of occurrence, Braden score, length of hospital stay, and the specifics of PI occurrence were factors influencing the outcomes of out-of-hospital PIs.

### Sex

Female patients had a higher improvement rate for out-of-hospital PIs, while male patients were more likely to develop PIs and experience less favourable outcomes. In China, the ideal body fat percentage for adults is below 20% for men and below 25% for women. The subcutaneous fat layer provides effective protection for the body’s circulatory system, helping to reduce the incidence of PIs, which may explain the better outcomes observed in female patients. PIs can occur in all individuals, and their prevention and treatment are equally important. With advancing age, the skin of older adult patients undergoes significant physiological changes, including gradual moisture loss in the stratum corneum, increased epidermal pH, and weakened barrier function (13). These changes collectively elevate the risk of PIs in the older adults. Therefore, it is particularly important to implement measures that protect skin health in this population to prevent PIs.

### PI characterization

Less than 1% of patients were from plastic surgery or wound care departments, indicating limited specialised involvement. General awareness of PI among patients and their families remains relatively low. Many do not fully understand the severity or preventive measures associated with PI and often overlook its potential risks, which can result in delayed treatment. It is recommended that hospitals establish dedicated PI consultation clinics or specialised nursing teams to offer accessible treatment and advisory services. Additionally, public awareness campaigns should be implemented to educate the community about PIs and their prevention.

Patients with tumours exhibited poorer outcomes in PI management. The healing process in these individuals is often slow and complicated due to compromised immunity and nutritional deficiencies, with some cases resulting in non-healing wounds or recurrent infections. For this vulnerable group, a personalised and comprehensive treatment plan should be developed, incorporating nutritional support and psychological care. These interventions can help strengthen overall immunity and resilience, thereby improving treatment outcomes and enhancing quality of life.

Trauma/orthopedic patients exhibited a higher odds ratio for PI healing compared to other disease categories. This finding should be interpreted with caution, as it may be influenced by several factors. These patients typically undergo surgical interventions and postoperative rehabilitation, which may improve their mobility and offloading capacity during hospitalization. In addition, the relatively longer hospital stays in orthopedic patients may provide a more extended treatment window for PI healing. However, residual confounding from unmeasured variables such as baseline functional status or specific orthopedic interventions cannot be ruled out.

### Braden score

The lower the Braden score, the higher the risk of developing PI. Our results indicate that this score is also associated with PI outcomes. Patients with lower risk scores tend to have better outcomes because they generally have better overall health and a higher capacity for self-repair, which facilitates recovery. In contrast, no statistically significant differences were observed in outcomes among patients categorised as very high or extremely high risk, although these groups consistently exhibited poorer prognoses. Such patients often present with complex medical conditions, including multiple comorbidities and complications, which substantially hinder PI recovery.

For these high-risk individuals, PI treatment is particularly challenging and demands more refined, personalised care strategies. However, due to the unpredictable nature of their conditions and the intricacies of treatment, these strategies may not always yield optimal results. It is essential for healthcare professionals to prioritise the use of the Braden score in PI risk assessment, enabling early identification of high-risk patients and the implementation of targeted preventive interventions. For those who have already developed PI, individualised treatment plans should be tailored to their Braden score and specific clinical profiles. Patients in the very high and extremely high-risk categories require increased attention and resource allocation, along with strengthened multidisciplinary collaboration and comprehensive care approaches to improve PI outcomes.

### Out-of-hospital PI occurrence

We found that, as the duration since PI onset increases and the extent of PI becomes more widespread, outcomes for older adult patients with out-of-hospital PI tend to deteriorate. This population often experiences reduced mobility and diminished independence, making them more vulnerable to prolonged pressure exposure. The widespread nature of PI contributes to extensive skin and tissue damage, elevating the risk of infection and complicating care, which collectively lead to poorer prognoses. Additionally, older adult patients frequently delay seeking medical attention, exacerbating the severity of their condition.

Current data show that aside from Stage 1 PI, a significant proportion of cases were deep tissue injuries (DTI) present on admission. This may stem from inadequate early intervention in out-of-hospital settings. DTI represents damage at the bone-muscle interface due to pressure and/or shear force and, while potentially reversible with timely offloading and supportive care, indicates a substantial degree of soft tissue compromise at the time of admission. In this cohort, these injuries were associated with higher clinical management complexity. Research has shown that in home care settings, up to 75% of PI care is provided by family members or caregivers who lack professional nursing knowledge (14). These non-professional caregivers primarily focus on daily living and emotional support, but often fall short in delivering clinical-grade nursing care, contributing to increased PI incidence (15) and negatively impacting outcomes.

This underscores the need to not only enhance hospital staff training in PI prevention and management, but also to extend educational efforts to family caregivers. Providing regular training, personalised guidance, and ongoing support can strengthen their caregiving competencies and significantly reduce the incidence and severity of out-of-hospital PI.

### Length of hospital stay

As hospital stay length increases, outcomes for older adult patients with PI tend to improve. However, when the hospital stay exceeds 1 month, there is no significant difference in outcomes compared to those with a stay of less than 1 week. The subjects in our study were older adult patients transferred to a tertiary hospital. Our hospital has a more professional wound and stoma care team, along with advanced medical equipment, which enables more comprehensive and systematic treatment and care for older adult patients. With longer hospital stays, patients benefit from continuous professional wound management, drug therapy, nutritional support, and other interventions that promote PI healing.

However, PI treatment remains limited by various factors. Once PI reaches a stage that is difficult to heal, even extended hospitalisation may not improve the outcome. In clinical practice, treatment and hospitalisation plans should be tailored to each patient’s specific condition to optimise medical resource use and enhance PI outcomes. It should also be acknowledged that the positive association between longer hospital stays and PI healing may partly reflect reverse causality—patients with more severe or slow-healing PIs may require extended hospitalization, and thus the longer stay is a consequence rather than a cause of the healing process. Therefore, while our findings suggest an association between hospital stay duration and healing outcomes, this relationship should not be interpreted as indicating that simply prolonging hospitalization will directly improve healing. The optimal length of stay should be determined based on individual patient conditions and clinical needs.

### Implications for the prevention and management of out-of-hospital PI in older adult patients

Our comprehensive data analysis reveals that our hospital has achieved notable success in managing older adult inpatients with out-of-hospital PIs. Despite an average hospital stay of only 10 days, the improvement rate among patients reached 70%, clearly reflecting the efficiency and professionalism of our hospital’s PI management and treatment. However, the number of older adult patients with such PIs remains substantial. This not only underscores the challenges of PI prevention and control in community and home-based nursing environments but also highlights the urgency of strengthening continuity of care between hospitals and the community to ensure seamless treatment and rehabilitation.

It is recommended that a systematic continuity of care programme be developed and implemented for older adult patients with out-of-hospital PIs. This should include long-term PI prevention education in primary healthcare institutions, pre-discharge education for older adult patients, home care guidance, regular follow-up and assessment, and other supportive measures to ensure patients receive professional PI management both before admission and after discharge. Such a programme may potentially reduce recurrence and readmission rates, although this remains to be tested in future prospective studies.

Public awareness of PI prevention should be enhanced through multi-channel and multi-format health education initiatives, such as brochures, lectures, and social media campaigns, particularly targeting older adult individuals and their caregivers to improve their ability to recognise PI risks and take preventive action. Collaboration and communication with surrounding nursing homes should also be strengthened. Through training sessions, workshops, and other formats, advanced concepts and techniques for PI prevention and management can be disseminated to improve the professional competence and practical skills of nursing home caregivers, thereby establishing an integrated PI prevention and control network within and beyond the hospital. Several limitations of this study should be acknowledged. First, this was a single-center retrospective study conducted at one tertiary hospital in Zhejiang Province, China, which may limit the generalisability of our findings to other settings or populations. Second, the binary outcome classification (healed vs. non-healed) combined “improved”, “no progress”, and “worsened” into a single category. While this approach aligns with previous large-sample retrospective studies and reflects the clinically meaningful endpoint of complete healing, we acknowledge that it simplifies the diverse healing trajectories observed in clinical practice. Future prospective studies using continuous assessment tools such as the PUSH score may provide more refined outcome evaluations. Third, several clinically important confounders known to influence PI healing—including nutritional status (albumin/BMI), infection, cognitive impairment, mobility level, and ICU admission—were not captured in our database, which relied on routinely collected clinical data not originally recorded for research purposes, and this may have resulted in residual confounding. However, the Braden score, which encompasses dimensions of nutrition, activity, and mobility, was included in the multivariable model and partially adjusted for these factors. Fourth, we did not include in-hospital mortality or palliative discharge data, which could have provided additional context regarding outcomes in this frail older adults population. Finally, the lack of post-discharge follow-up limited our ability to assess long-term healing and recurrence rates. Despite these limitations, the large sample size and standardized data collection and verification procedures strengthen the reliability of our findings. In addition, we did not include in-hospital mortality or palliative discharge data in our outcome analysis. Given the advanced age and frailty of our study population, these outcomes are clinically relevant and may have provided additional context for understanding the full spectrum of patient prognoses. However, the primary objective of this study was to identify factors associated with PI healing among surviving patients during hospitalization, and we therefore focused on wound healing outcomes rather than overall survival or discharge disposition. Nevertheless, we acknowledge this as a limitation and recommend that future research incorporate these important endpoints to provide a more comprehensive assessment of outcomes in this vulnerable population.

## Conclusion

This study was organised and implemented by a highly professional wound and stoma care team at a tertiary hospital, ensuring the reliability and scientific rigor of the assessment results. Given the existing gaps in PI prevention and management in other hospitals and nursing homes, professional teams and tertiary hospitals should actively leverage their expertise. Targeted support services, such as skills training, on-site guidance, and resource sharing, can be provided to strengthen PI prevention awareness and management capabilities in nursing homes. These efforts not only improve the quality of life of older adult residents and reduce the incidence and severity of PI but also promote the integration of medical and older adults care services. By advancing this collaborative model, a more comprehensive and efficient integrated medical-nursing service system can be established, contributing to social harmony and the wellbeing of the older adults population.

## Data Availability

The raw data supporting the conclusions of this article will be made available by the authors, without undue reservation.
